# Impact of Semaglutide on Hippocampal Injury in a Streptozotocin-Induced Model of Alzheimer’s Disease

**DOI:** 10.3390/biomedicines14061257

**Published:** 2026-05-31

**Authors:** Alla V. Stavrovskaya, Anastasia K. Pavlova, Dmitry N. Voronkov, Artem S. Olshanskiy, Alexandr S. Romanenko, Evgenia N. Fedorova, Anastasia V. Simonenko, Vladimir S. Sukhorukov, Sergey N. Illarioshkin

**Affiliations:** Russian Center of Neurology and Neurosciences, Moscow 125367, Russia; pav_nastasya@mail.ru (A.K.P.); sukhorukov@neurology.ru (V.S.S.);

**Keywords:** streptozotocin, semaglutide, Alzheimer’s disease, hippocampus

## Abstract

**Background**: Glucagon-like peptide-1 receptor (GLP1R) agonists, particularly semaglutide, show neuroprotective effects in genetic models of Alzheimer’s disease (AD). However, their delayed and long-term effects in sporadic AD, such as the intracerebroventricular streptozotocin (STZ) injection, remain insufficient. It is unclear how long the effects of GLP1R agonists persist after discontinuation and whether a single course can suppress progressive neurodegeneration. This study aimed to evaluate the delayed effects of semaglutide administration on morphological changes in neurons and glial cells in the hippocampus associated with cognitive impairment in an STZ-induced rat model of AD. **Methods**: Rats received bilateral intracerebroventricular STZ injections (3 mg/kg) followed by a 5-week course of intraperitoneal administration of semaglutide (0.1 mg/kg, every other day), and were euthanized 60 days after discontinuation of semaglutide administration. Immunomorphological methods were used to detect neuronal, astrocytic and microglial alterations. A novel object recognition test was performed to assess behavioral effects. **Results**: STZ-treated animals demonstrated cognitive impairments, ventriculomegaly, a significant increase in p-tau protein fluorescence intensity (*p* = 0.02), a decrease in CA1–CA3 field area (by 23%, *p* = 0.008), and reduced hippocampal neuronal density. Decreases in TOMM20 (mitochondrial marker) and synaptophysin levels were accompanied by significant glial activation in the hippocampal CA3 field. Semaglutide administration significantly reduced the enlarged ventricular lumen (by 43.5%), decreased p-tau fluorescence intensity, reduced vimentin-positive reactive astrocytes (by 68.4%), and increased synaptophysin fluorescence intensity. Furthermore, it reduced microglial activation (decreasing IBA1 cell density and elongation) and alleviated the disrupted AQP4 distribution. However, semaglutide did not completely halt the neurodegenerative process and showed no effect on the number of doublecortin-positive cells in the dentate gyrus. **Conclusions**: Hippocampal changes assessment revealed that course administration of semaglutide exerts prolonged effects, attenuating the severity of pathomorphological alterations and behavioral changes in a sporadic AD model after drug discontinuation.

## 1. Introduction

Alzheimer’s disease (AD) is characterized by the accumulation of pathological protein aggregates, including extracellular amyloid-β (Aβ) plaques and intracellular neurofibrillary tangles composed of hyperphosphorylated tau protein (p-tau), leading to neurodegeneration and cognitive decline [1].

The amyloid cascade hypothesis remains one of the leading concepts in AD research, proposing that Aβ accumulation is a key trigger of tau pathology and neurodegeneration [2]. Despite extensive efforts, the development of new agents targeting amyloid cascades based on genetic models of AD has yielded limited efficacy.

Moving beyond the primary role of amyloid-β accumulation, emerging theories suggest that AD develops due to impaired energy metabolism and reduced glucose uptake. Postmortem and in vivo studies demonstrate reduced cerebral glucose metabolism in AD [3,4,5]. Notably, fluorodeoxyglucose positron emission tomography (FDG-PET) reveals hypometabolism in vulnerable brain regions even in individuals with mild cognitive impairment or preclinical AD [6,7].

Several authors associate impaired cerebral glucose uptake in AD with insulin receptor (IR) dysfunction and dysregulation of insulin signaling [8], which, according to the “type 3 diabetes” hypothesis, underlie AD pathogenesis and lead to impaired neural tissue energy metabolism, including disruptions in neuro-gliovascular interactions. Insulin-dependent glucose regulation in the brain appears to play a critical role at the level of local neural networks [9]. IR deficiency results in impaired gliotransmission, mitochondrial dysfunction, and increased reactive oxygen species (ROS) production [10,11]. These findings suggest that impaired glucose utilization may represent an early feature of AD rather than merely a consequence of neurodegeneration.

Among promising agents for AD therapy, significant attention is focused on the neuroprotective properties of glucagon-like peptide-1 (GLP1) receptor agonists. GLP1, produced by neuroendocrine L-cells of the intestine, enhances insulin release from pancreatic β-cells at the systemic level, inhibits glucagon secretion, and mediates behavioral effects associated with hunger and satiety [12]. Beyond its role in systemic glucose homeostasis, intestinal GLP1 activates relevant brain systems by stimulating GLP1-ergic neurons of the nucleus tractus solitarius (NTS) via vagal afferents, as well as crossing the blood–brain barrier (BBB) at circumventricular organs [13].

The highest expression of GLP1 receptors (GLP1R) in the brain is predominantly found in structures linked to reward mechanisms and feeding behavior [14,15], which are densely innervated by NTS neurons: the arcuate, paraventricular, and dorsomedial nuclei of the hypothalamus, as well as the nucleus accumbens, septum, and ventral tegmental area of the midbrain. Despite the relatively high levels of GLP1R and the number of GLP1R-expressing neurons in the cortex and hippocampus of both animals and humans [16,17], these structures receive fewer GLP1-ergic projections from the NTS compared to hypothalamic regions. However, they may serve as targets for intestinal GLP1 and its pharmacological analogs, which enter the brain primarily through circumventricular organs upon systemic administration. Additionally, GLP1R are found in microglia, but data on astrocytes remain conflicting [14,15]. Notably, the distribution of GLP1R broadly aligns with brain regions most vulnerable in AD.

Pharmacological activation of GLP1R has been shown to affect GABAergic tonic currents in cortical [18], hypothalamic [19], and hippocampal neurons [20]. GLP1 also acts locally on insulin cascades in brain structures, for example, by stimulating insulin secretion with neurogliaform cortical interneurons and hypothalamic neurons [18,21]. Beyond regulating insulin sensitivity, GLP1R activation is associated with the modulation of EGFR and BDNF growth factors, which stimulate cell proliferation, differentiation, and growth.

Currently, GLP1R agonists are approved for clinical use in glycemic and body weight control. Developed recombinant GLP1 analogs differ in their ability to cross the BBB and their tissue-specific metabolic rates, which depend on the activity of tissue proteases toward these peptides [22]. Among existing agents, semaglutide stands out in terms of CNS bioavailability and half-life. Recent clinical trials [23] in patients with early AD failed to demonstrate clinical efficacy in slowing cognitive decline, despite inducing positive shifts in neuroinflammatory and AD-related fluid biomarkers. Given the extensive experimental data supporting the neuroprotective potential of GLP1R agonists, this underscores the necessity of evaluating their effects in models of sporadic AD.

The mechanisms underlying the neuroprotective effects of GLP1R agonists remain incompletely understood and include reduced pro-inflammatory activation of microglia and astrocytes, restoration of neuro-glio-vascular interactions, improved insulin sensitivity and energy metabolism regulation, with potential additional effects on neurogenesis [24,25,26].

Although promising results have been demonstrated for the neuroprotective properties of GLP1R agonists in models of neurodegenerative diseases, existing literature provides insufficient data on semaglutide’s specific effects on the progression and advanced stages of Alzheimer’s pathology in models of sporadic AD. Key outstanding questions include the durability of GLP1R agonist effects after treatment discontinuation and the degree to which these agents can suppress progressive neurodegenerative processes.

Intracerebroventricular injection of STZ is used to model sporadic AD in rats and is considered, among other things, a reproduction of the cerebral insulin-resistant state. When administered systemically, STZ selectively damages pancreatic islet β-cells; however, when administered intracerebroventricularly, as shown in our previous works [27], primarily affects tanycytes and hypothalamic structures adjacent to the third ventricle that are involved in systemic and local glucose regulation. STZ-induced reduction in cerebral glucose uptake is irreversible and leads to progressive changes in nervous tissue, associated with IR desensitization, impaired insulin production, dysfunction of glucose transporters GLUT1-4, and damage to tanycytes serving as glucose sensors [28]. Current evidence does not support the predominance of any particular mechanism of STZ action.

In animals receiving intracerebroventricular injections of STZ, neuronal damage, white matter degeneration, gliosis, and amyloid-β accumulation are observed [29,30,31]. The literature highlights the vulnerability of the hippocampus to STZ effects, particularly the neurons of the CA1-3 fields, which aligns with findings in human AD. Following injection of STZ, the hippocampus of animals exhibits increased synthesis of pro-inflammatory cytokines, activation of astrocytes and microglia, and reduced expression and phosphorylation of insulin receptor substrate 1 (IRS1) and other molecules involved in insulin signaling cascades [30,32]. Furthermore, STZ has been shown to induce tau protein hyperphosphorylation, a hallmark of AD that leads to the formation of neurofibrillary tangles [30]. Comparative studies of STZ and genetic models of AD have demonstrated similar impairments in IR/IGF signaling cascades and neuroinflammatory changes [33].

This study aimed to evaluate the delayed effects of semaglutide, a glucagon-like peptide-1 receptor agonist, on memory deficits (novel object recognition test), ventriculomegaly, neurodegeneration and neurogenesis (NeuN, doublecortin), tau-protein phosphorylation (p-Tau Thr217), and glial activation (GFAP, vimentin, AQP4 for astrocytes; IBA1 for microglia), as well as mitochondrial and synaptic alterations (TOMM20, synaptophysin) in a STZ-induced Alzheimer’s disease rat model.

## 2. Materials and Methods

### 2.1. Animals

The study was performed on 34 Wistar male rats, aged 3.5 months at the start of the experiment. The animals were randomly divided into two groups: one received intracerebroventricular injections of STZ, and the other received 0.9% NaCl solution. The animals were housed under standard climate-controlled conditions, with a 12 h light/dark cycle, with food and water ad libitum.

### 2.2. Stereotaxic Surgery

For anesthesia during intraventricular injections, Zolethyl 100 (Valdepharm, Val-de-Reuil, France) at a dose of 30 mg/kg and Xyla (Interchemie Werken “De Adelaar” B.V., Waalre, The Netherlands) at a dose of 3 mg/kg were administered intramuscularly. The intracerebral injections were performed using a stereotaxic instrument (RWD Life science Co., Shenzhen, China) and a Hamilton syringe 26 g/51 mm needle (Hamilton Bonaduz AG, Bonaduz, Switzerland) with the following coordinates: AP = −0.8; L = 1.5; V = 3.5 (in accordance with “The Rat Brain in Stereotaxic Coordinates” atlas [34]). Each animal received bilateral intraventricular injections of 5 μL streptozotocin (streptozocin) solution (3 mg/kg, freshly diluted and kept in the dark due to light instability) or 0.9% NaCl. Average weight of animals at the date of surgery: 348.6 ± 3.6 g (mean ± SEM). Administration of STZ was associated with a baseline mortality rate of approximately 3%. These lethal outcomes occurred exclusively within the first 3–5 days post-surgery due to acute drug-induced toxicity, and were completely excluded from all subsequent analyses.

### 2.3. Semaglutide Administration

Seven days after stereotactic surgeries [35,36], animals from both groups received intraperitoneal injections of semaglutide solution (CAS Number: 910463-68-2, GEROPHARM LLC, Moscow, Russia) at a dose of 0.1 mg/kg [37,38,39,40,41] every other day for 5 weeks (16 injections). The remaining animals similarly received intraperitoneal injections of 0.9% NaCl. This resulted in the formation of four groups: (1) control (saline, *n* = 8); (2) control + semaglutide (saline + Sm, *n* = 8); (3) streptozotocin (STZ, *n* = 9); (4) streptozotocin + semaglutide (STZ + Sm, *n* = 9).

### 2.4. Behavioral Testing

The novel object recognition (NOR) test was performed before euthanizing the animals. Habituation was performed the day before the first test session by placing each animal in the test arena for 5 min in the absence of any objects. On the first day of testing, animals were placed in a 90 × 90 cm arena with 42 cm high walls containing two identical objects (cubes) symmetrically positioned at equal distances from the walls. The duration of the animal’s interaction with the objects was recorded. On the second day, one “familiar” object was replaced with a novel object—a cone. Each testing session lasted 5 min. Discrimination Index (DI) was calculated as (Novel Object Exploration Time/Total Exploration Time)—(Familiar Object Exploration Time/Total Exploration Time). After each test, the arena floor, walls and objects were thoroughly cleaned with 70% ethanol solution.

### 2.5. Immunomorphological Study

Animals were euthanized 60 days after discontinuation of semaglutide administration. Rats were decapitated using a guillotine; brains were extracted, sectioned and fixed for 24 h in 4% phosphate-buffered formalin (pH 7.2–7.4). Samples were immersed in 30% sucrose solution until they sank, frozen in TIssue Tek O.C.T. medium (Sakura Finetek Inc., Torrance, CA, USA), and 10-μm-thick frontal cryostat sections were prepared. Nissl staining was performed using 0.1% cresyl violet solution and mounted in DPX medium.

Antigen retrieval for immunofluorescence staining was performed by heating in citrate buffer (pH 6.0) for 15 min at 96–98 °C in a steam cooker, after which sections were cooled and rinsed with distilled water. Phosphate-buffered saline (0.01 M, pH 7.2–7.4) containing 0.05% Triton X-100 was used for subsequent washing used. Prior to primary antibody application, sections were incubated for 20 min with 1% bovine serum albumin to reduce nonspecific binding. Sections were incubated with primary antibodies for 18 h at RT, washed and then incubated with corresponding secondary antibodies for 3–4 h in a humidity chamber.

The following primary antibodies were used to identify cellular types: anti-neuronal marker NeuN (Abcam, Cambridge, UK, ab177487, Rabbit, monoclonal, ab104224, Mouse, monoclonal), anti-glial fibrillary acidic protein GFAP (Abcam, Cambridge, UK, ab207165, Rabbit, monoclonal; ab279290, Mouse, monoclonal—were used for imaging only purpose in double-staining with AQP4), and anti-microglial marker IBA1 (Abcam, Cambridge, UK, ab178847, Rabbit, monoclonal). Anti-doublecortin antibodies (DCX, GeneTex, Irvine, CA, USA, GTX134052, Rabbit, polyclonal) were used to detect neuronal precursors in the hippocampal subgranular zone. Phosphorylation of tau protein at threonine 217 (pTau) was assessed (Invitrogen, Carlsbad, CA, USA, cat. #44-744, Rabbit, polyclonal). Antibodies against glial and endothelial proteins vimentin (Vim, Abcam, Cambridge, UK, ab92547, Rabbit, monoclonal) and aquaporin-4 (AQP4, Merck KGaA, Darmstadt, Germany, HPA-014-784, Rabbit, polyclonal) were applied, as well as antibodies to synaptic marker protein synaptophysin (SYP, Abcam, Cambridge, UK, ab32127, clone YE269, Rabbit, monoclonal) and mitochondrial outer membrane marker TOMM20 (Abcam, Cambridge, UK, Mouse, ab283317 (EPR15581-39), monoclonal). Binding was detected using corresponding secondary antibodies: Alexa Fluor 488-conjugated anti-rabbit immunoglobulins (Abcam, Cambridge, UK, ab150077, goat) and Alexa Fluor 594-conjugated anti-mouse (Invitrogen, Carlsbad, CA, USA, A-11032, goat) and anti-rabbit (Abcam, Cambridge, UK, ab150080, goat) immunoglobulins. Antibodies were applied according to manufacturer-recommended protocols. Sections were mounted in FluoroShield medium containing DAPI for nuclear counterstaining.

### 2.6. Image Analysis

Specimens were documented using Nikon SMZ18 and Nikon Eclipse Ni-U fluorescent microscopes (Nikon Corporation, Tokyo, Japan) using an appropriate filter set. Quantitative analysis was performed using Nikon NIS Elements BR ver. 4.0 or ImageJ ver. 1.54 software. The researcher performing the morphometry measurements was blinded to the experimental group of analyzed samples.

Morphometric analysis utilized serial sections from the anterior third of the hippocampus (6–12 sections taken at 200 μm intervals were used in each analysis), with assessments performed in both left and right hemispheres. Staining intensity was evaluated in manually outlined regions of interest (ROIs) based on mean brightness (8-bit grayscale values). Hippocampal area measurements were performed in its rostral third using NeuN-stained specimens for detecting a marker protein, with ROIs manually segmented. The area of the third ventricle lumen was measured on the same rostro-caudal levels on Nissl-stained sections. Neuronal density was assessed in CA3 field images acquired at 20× magnification by manually counting NeuN-positive cells with visible nuclei. Similarly, DCX-positive cells in the subgranular zone were counted at 20× magnification, and the granule cell layer length of the dentate gyrus (along its inferior border) was measured to calculate the number of DCX-positive cells per 1 mm. For assessment of TOMM20 staining NeuN-positive cells (50 randomly selected CA3 neurons from 5 sections per animal) in CA3 field on images obtained using objective 60× were manually outlined using a graphic tablet and nuclear areas were excluded from analysis. Microglial density and GFAP-positive area were quantified using local threshold (ImageJ implemented Otsu algorithm) segmentation. IBA1-positive cells with detectable nuclei were counted per field of view (0.16 mm^2^). GFAP-positive area was expressed as a percentage of the field of view. IBA1-positive cells processes were manually outlined and 25–30 randomly selected cells per animal were used for determining aspect ratio shape factor—to determine elongation of microglia and their transformation to reactive state.

Statistical analysis was performed using Statistica and GraphPad Prism 8.0. Due to the exploratory nature of the study, the sample size was determined in accordance with the Resource equation method and in line with similar publications. Outliers were identified using ROUT method (Q = 1%). Shapiro–Wilk test for normality testing was performed. Mean values per animal were calculated and used as the primary statistical unit. Between group comparisons were made using the Kruskal–Wallis test followed by Dunn’s post hoc test. In case of normality test passed for all groups ANOVA with Tukey post hoc test or Brown-Forsythe ANOVA with post hoc unpaired *t*-test with Welch correction were used.

## 3. Results

**Figure 1:**
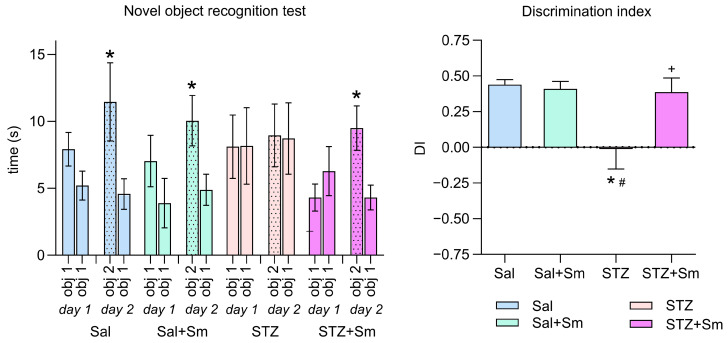
Time spent by animals to explore an object in the Novel object recognition test. day 1—first test session (identical objects—“obj 1”); day 2—second test session, “obj 2” indicates novel object (dot-filled bars). *—*p* < 0.05 compared to all timings in this group, 2-Way ANOVA, Dunnett’s test. Mean ± SD. Discrimination index, 1-Way ANOVA, Tukey test. Mean ± SEM “*”—*p* < 0.05 vs. saline (control) group; “#”—*p* < 0.05 vs. saline + Sm group; “+”—*p* < 0.05 vs. STZ group. Group designations hereafter: Sal—saline (control), Sal + Sm—saline + semaglutide, STZ—streptozotocin, STZ + Sm—streptozotocin + semaglutide.

During the NOR test on day one, no significant differences in the time spent exploring identical objects were observed among rats in all groups. On day two, control animals spent significantly more time exploring the novel object compared to the familiar one (11.46 ± 2.9 vs. 4.57 ± 1.1 s, *p* < 0.05). In contrast, rats receiving STZ alone showed no difference in interaction time between the two objects (Figure 1), indicating memory impairments. Animals treated with semaglutide alone or semaglutide combined with STZ also demonstrated significantly longer exploration time of the novel object. Discrimination index was significantly lower in the STZ-treated group, in comparison with control groups and STZ treated animals received semaglutide (Figure 1).

**Figure 2:**
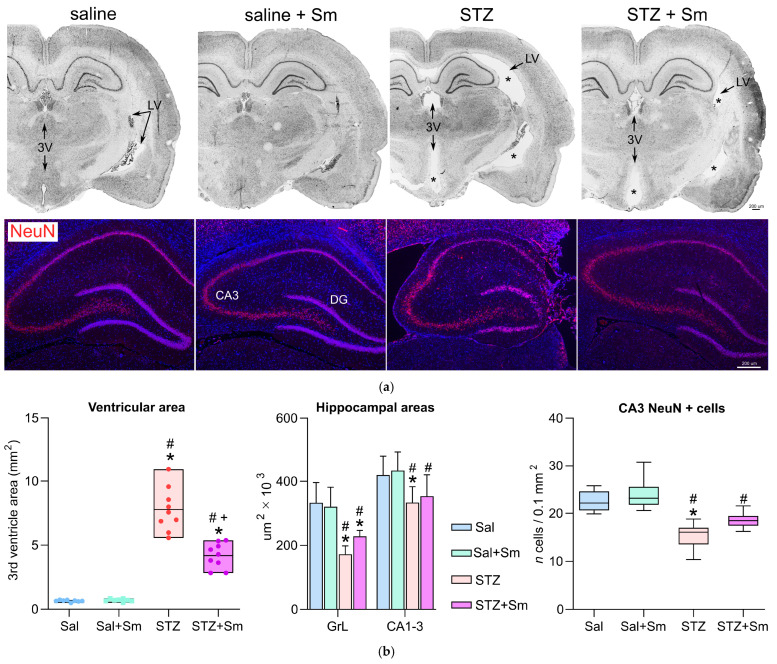
(**a**) Overall hippocampal alterations in the STZ model and attenuation of these changes under the influence of semaglutide (Sm). Nissl staining, upper panel and NeuN (shown in red) immunofluorescence staining—lower panel (DAPI—blue). Asterisk marks enlarged ventricular spaces, LV—lateral ventricle, 3V—third ventricle lumen, CA3—Cornu ammonis CA3 area, DG—dentate gyrus of hippocampus, GrL—granular layer, fi—fimbria hippocampi. (**b**) “Ventricular area”—3rd ventricle area (Brown-Forsythe ANOVA, post hoc unpaired *t*-test with Welch correction); “Hippocampal areas”—area of hippocampal regions (GrL—granular layer, CA1–CA3 pyramidal layer of CA1–CA3 area, 2-Way ANOVA, post hoc Tukey test), “CA3 NeuN^+^ cells”—density of neurons in CA3 field of hippocampus (Kruskal–Wallis test, post hoc Dunn’s test). “*”—*p* < 0.05 vs. saline (control) group; “#”—*p* < 0.05 vs. saline + Sm group; “+”—*p* < 0.05 vs. STZ group. Bar plots are represented as Mean ± SD. Ventricular area graphs are represented as Mean and min–max range with dots as single animal means. Hereafter, box-and-whisker plots represent the median (horizontal line), quartiles (box edges), and whiskers (1.5 times the interquartile range).

Intracerebroventricular STZ administration caused marked enlargement of the ventricular system, deformation, and atrophy of the rostral hippocampal regions. Neurodegenerative changes were observed in the hippocampus (Figure 2a).

To assess changes in the ventricular system, the cross-sectional area of the third ventricle was measured (Figure 2b) to minimize measurement bias associated with the complex anatomical shape of the lateral ventricles. Furthermore, alteration in the third ventricle reflected abnormalities in CSF dynamics rather than ventriculomegaly secondary to hippocampal atrophy. We observed a statistically significant increase in the third ventricle area in STZ-induced animals, whereas semaglutide treatment led to a significant reduction in ventricular lumen in the STZ + Sm group (median value decreased by 43.5% in comparison to the STZ group, *p* < 0.001 (Brown-Forsythe ANOVA, post hoc unpaired *t*-test with Welch correction), although mean area values did not reach the control level.

Assessment of the granular layer area (Figure 2b) in the dentate gyrus using NeuN staining revealed a 46% reduction in the STZ-only group (*p* < 0.0001, ANOVA, Tukey’s post hoc test) and a 28% reduction in the STZ + Sm group compared to the control group receiving semaglutide alone. Similar results were obtained when compared to the control group. However, the difference between the STZ and STZ + Sm groups was not statistically significant. The pyramidal layer in CA1-3 fields was less affected than the dentate gyrus granular layer. Specifically, the CA1-3 field area decreased by 23% (*p* = 0.008) in the STZ group and 18% in the STZ + Sm group compared to the semaglutide-only group. The observed reduction in hippocampal area may be associated with its deformation due to ventricular enlargement and subsequent atrophy of hippocampal structures, especially noticeable in the fimbria of the hippocampus (Figure 2a). Additionally, signs of neuronal degeneration were noted, as CA3 field neuronal density was significantly reduced (*p* < 0.001) in STZ-treated animals.

**Figure 3:**
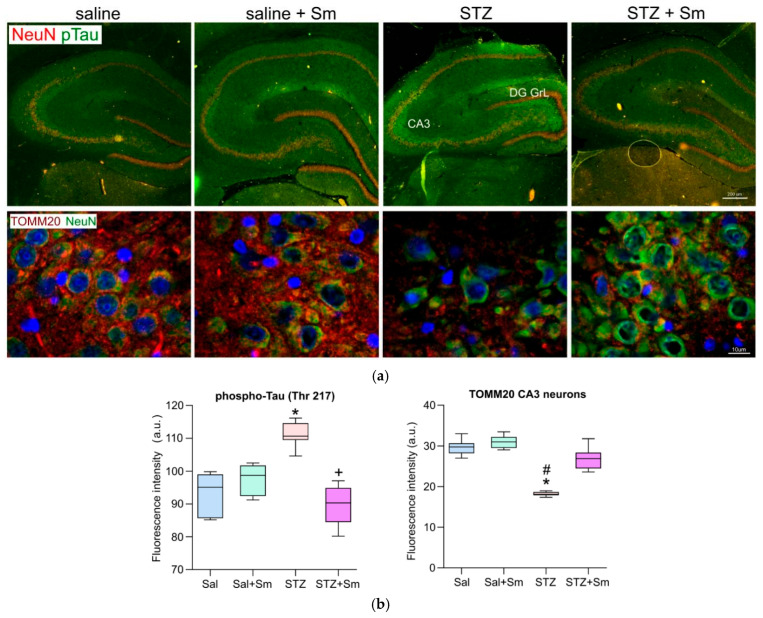
(**a**) Immunofluorescent staining of phosphorylated tau protein (pTau-Thr 217 in green on the upper panel, NeuN—red) in hippocampus and outer mitochondrial membrane marker (TOMM20 in red on the lower panel, NeuN—green, DAPI—blue). (**b**) “phospho-Tau (Thr 217)”—Intensity of fluorescence for pTau in the CA3 field of hippocampus; “TOMM20 CA3 neurons”—Intensity of fluorescence for TOMM20 in the CA3 field of hippocampus. Kruskal–Wallis test, post hoc Dunn’s test. “*”—*p* < 0.05 vs. saline (control) group; “#”—*p* < 0.05 vs. saline + Sm group; “+”—*p* < 0.05 vs. STZ group.

Neuronal changes under STZ influence were accompanied by a significant increase (*p* = 0.02, Kruskal–Wallis test with Dunn’s post hoc test) in phosphorylated tau protein (Thr 217 residue) immunofluorescence intensity (Figure 3a,b); these changes likely contribute to neuronal damage. The STZ + Sm group exhibited a significant decrease in this parameter (*p* < 0.01 vs. the STZ group).

The immunofluorescence intensity of TOMM20 (Figure 3a,b) in neuronal somata was significantly reduced in the STZ-treated group (*p* < 0.001, Kruskal–Wallis test with Dunn’s post hoc test). This alteration suggests a reduction in mitochondrial mass. In contrast, no significant differences were observed between the STZ + Sm group and the control groups.

**Figure 4:**
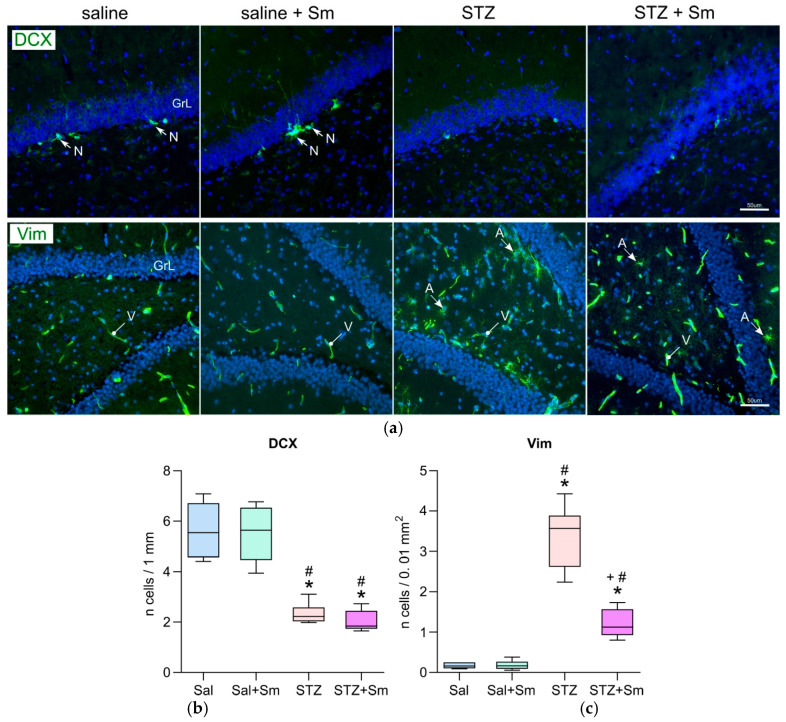
(**a**) Localization of DCX-positive cells and vimentin (Vim) localization in the dentate gyrus upon streptozotocin (STZ) administration and after semaglutide (Sm) treatment. “N” indicates DCX-positive immature neuronal cells, “A” indicates Vim-positive astrocytes, “V” indicates Vim-positive endothelial cells (vessels). DAPI—blue. (**b**) “DCX”—Mean number of DCX-positive cells per mm length of the subgranular zone. (**c**) “Vim”—means the number of Vim-positive astrocytes per hilus area. Kruskal–Wallis test, post hoc Dunn’s test “*”—*p* < 0.05 vs. saline (Sal) (control) group; “#”—*p* < 0.05 vs. saline + semaglutide (Sal + Sm) group; “+”—*p* < 0.05 vs. STZ group.

Impairment of neurogenesis in the dentate gyrus was also induced by STZ. Quantification of DCX-positive cells in the subgranular zone and granular layer of the dentate gyrus was performed (Figure 4a,b). The analysis demonstrated that STZ caused a more than twofold reduction in these cells (*p* = 0.01, Kruskal–Wallis test with Dunn’s post hoc test). This decrease was observed in both the STZ and STZ + Sm groups compared to controls.

Vimentin staining (which increases in immature and activated astroglia) revealed a significantly raised number (*p* < 0.01, Kruskal–Wallis test with Dunn’s post hoc test) of Vim-positive astrocytes that were nearly absent in control groups (Figure 4a). The STZ + Sm group demonstrated a statistically significant reduction in the number of these cells by 68.4% compared to animals treated with STZ alone (Figure 4c).

**Figure 5:**
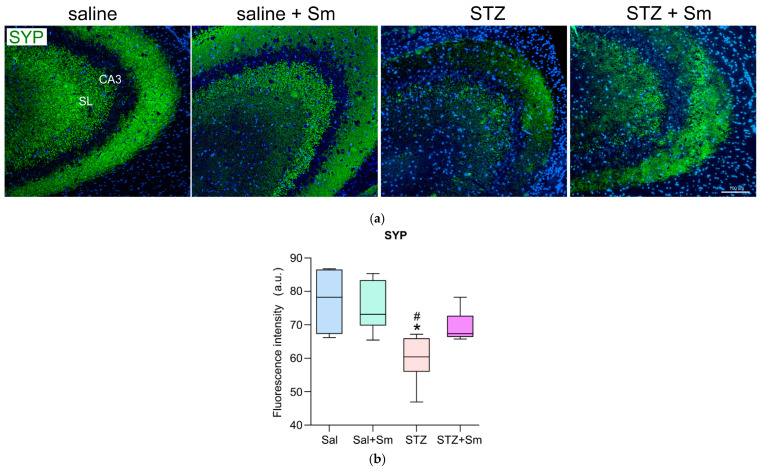
(**a**) Synaptophysin (SYP, shown in green) localization in the CA3 area of the hippocampus upon streptozotocin (STZ) administration and after semaglutide (Sm) treatment. “SL” indicates the stratum lucidum layer. DAPI—blue. (**b**) SYP fluorescence staining intensity. Kruskal–Wallis test, post hoc Dunn’s test “*”—*p* < 0.05 vs. saline (control) group; “#”—*p* < 0.05 vs. saline + Sm group.

The detrimental influence of STZ on hippocampal neurons was also reflected in synaptophysin (SYP) staining (Figure 5a,b). This effect was especially pronounced in the stratum lucidum layer on the inner border of the CA3 pyramidal layer, predominantly formed by mossy fibers.

STZ-treated animals showed a significant decrease in SYP staining intensity. The STZ + Sm group exhibited no differences from controls.

**Figure 6:**
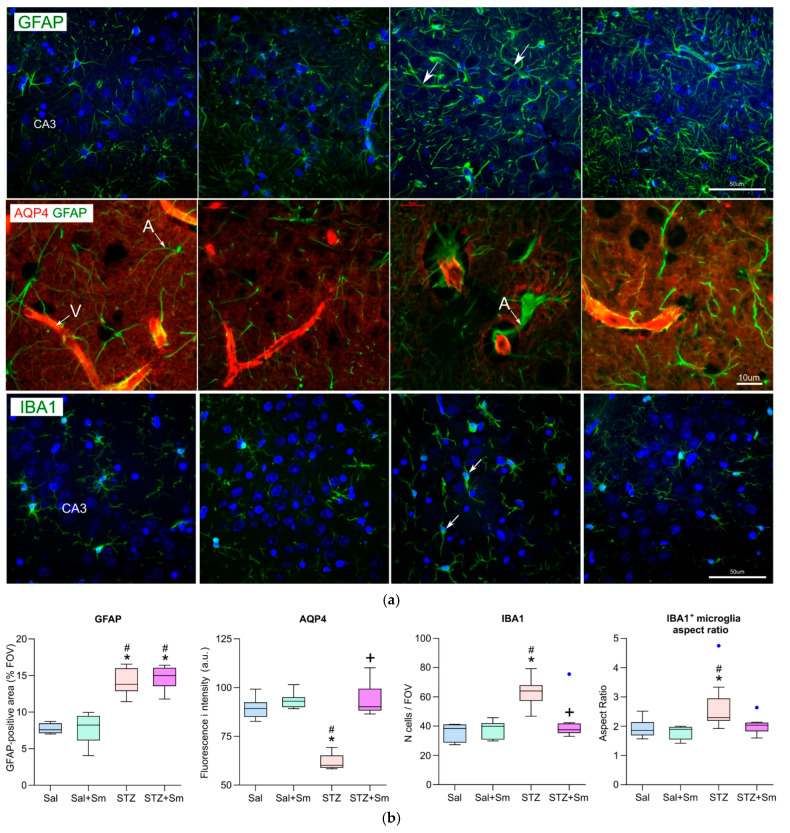
(**a**) Immunofluorescence detection of astroglial proteins GFAP (shown in green) and AQP4 (shown in red) and microglia marker IBA1 (shown in green) in the CA3 area of the hippocampus upon streptozotocin (STZ) administration and after semaglutide (Sm) treatment. DAPI—blue. (**b**) “GFAP”—relative area of GFAP-positive staining per field of view (%) in the CA3 field; “AQP4”—fluorescence staining intensity (a.u.) for AQP4 in the CA3 field; “IBA1”—cell density of IBA1-positive microglia (number of cells per field of view) in the CA3 field; “IBA1^+^ microglia aspect ratio”—morphometric shape factor (aspect ratio) reflecting elongation of IBA1-positive microglial cells. Kruskal–Wallis test, post hoc Dunn’s test “*”—*p* < 0.05 vs. saline (control) group; “#”—*p* < 0.05 vs. saline + Sm group; “+”—*p* < 0.05 vs. STZ group. Blue dot—outliers. Arrows on the upper GFAP panel indicate hypertrophied polarized astrocyte processes. Arrows on the IBA1 panel indicate elongated reactive glial cells with few processes. “A”—astrocyte processes, “V”—vessels.

STZ administration led to marked reactive changes in glial cells (Figure 6a,b). Specifically, a significant increase was observed in the area occupied by GFAP-positive astrocyte structures and in the density of IBA1-positive microglia in the CA3 field of the hippocampus. In the STZ-group, the microglial aspect ratio indicated a shift toward an elongated reactive phenotype.

Hippocampal astrocytes in the STZ-treated group exhibited pronounced polarization and thickened processes penetrating the pyramidal layer of the CA3 field (Figure 6a). Compared to other hippocampal layers, animals receiving STZ visually showed increased GFAP staining in the pyramidal cell layer. Semaglutide administration to STZ-treated animals did not significantly reduce the area occupied by astroglial processes; however, astrocytes in the STZ + Sm group appeared less hypertrophied and polarized. A significant decrease (by 41%, *p* = 0.023, Kruskal–Wallis test, post hoc Dunn’s test) in IBA1-positive microglial density was observed in the CA3 field of the STZ + Sm group compared to STZ alone. No significant differences from controls were detected in animals receiving semaglutide alone. The attenuation of microglia activation was also confirmed by changes in the shape of the cells.

Semaglutide promoted the restoration of microglial ramification, and in the STZ group, the microglial aspect ratio was significantly (*p* = 0.027, Kruskal–Wallis test, post hoc Dunn’s test) higher than in the control group, while in the STZ + Sm group, no differences with the control were shown.

Astrocyte activation was accompanied by altered AQP4 expression and distribution in the tissue. In control groups, AQP4 immunoreactivity was uniformly distributed throughout the neuropil, reflecting a high density of peripheral astroglial processes carrying water channels. Strong AQP4 staining was also detected in the vascular network. In the STZ-treated group, there was a preserved staining of the vascular wall, whereas AQP4 fluorescence intensity was reduced in the surrounding tissue. Furthermore, STZ administration led to abnormal distribution of AQP4 and increased staining around individual reactive astrocytes (Figure 6a). Overall, AQP4 fluorescence intensity was reduced in the STZ group. In the STZ + Sm group, AQP4 intensity matched that of controls and differed significantly from the STZ group (*p* = 0.0019, Kruskal–Wallis test with Dunn’s post hoc test), though individual astrocytes with disrupted AQP4 distribution in processes were still detected in the hippocampus.

Thus, STZ administration caused hippocampal neuronal damage, phosphorylated tau protein accumulation, astrogliosis with altered AQP4 distribution, and increased microglial reactivity. Animals receiving a delayed course of semaglutide after STZ administration demonstrated greater preservation of hippocampal structures. Furthermore, these animals exhibited lower p-tau immunofluorescence intensity. In STZ-treated animals, semaglutide showed a trend toward restoring synaptophysin levels in the hippocampal stratum lucidum layer. However, semaglutide did not normalize STZ-induced suppression of neurogenesis. Semaglutide also influenced pro-inflammatory glial reactivity: it normalized AQP4 levels in astrocytes and reduced microglial density, indicating attenuated neuroinflammatory processes. Notably, control animals receiving semaglutide alone showed no differences from the control group in the evaluated parameters.

## 4. Discussion

### 4.1. Relevance of the STZ Model to the AD Pathology

The behavioral testing data from STZ-treated animals obtained in this study are consistent with findings by other authors. Specifically, intracerebroventricular STZ administration causes progressive deterioration of performance in spatial memory and passive avoidance tests in mice with an increase in anxiety [31]. The authors associate STZ-induced cognitive impairments with elevated GSK3β expression and amyloid-β accumulation, which disrupt synaptic plasticity, damage granular layer neurons, and reduce dendritic spine density [31]. These observations are corroborated by our data showing altered synaptic protein levels in the CA3 field of the hippocampus in STZ-treated animals.

The behavioral changes identified in our study generally confirm previous reports of persistent synapsin-1 downregulation and memory deficits in the NOR test following intracerebroventricular STZ administration [36]. However, the same study demonstrated via Western blotting that increased tau protein phosphorylation was transient and decreased over time (21 days) post-STZ administration. Conversely, our STZ-treated group exhibited long-term persistence of all changes, aligning with reports of hyperphosphorylated tau accumulation in the hippocampus of STZ-treated rats [42]. The STZ model has also shown reduced levels of pre- and postsynaptic proteins, including SYP and PSD95, in the synaptosomal fraction [43], complementing our findings of decreased SYP immunostaining in the hippocampus of STZ-treated animals.

The general pathomorphological findings in the STZ-treated group—such as damage to the hippocampal fields, ventricular enlargement, and white matter degeneration—are consistent with the findings of other authors [28,44]. These macrostructural changes are likely driven by STZ-induced severe oxidative stress and mitochondrial dysfunction [44]. Specifically, STZ action has been shown to cause changes in membrane potential, reduced viability of mitochondria, degradation and fragmentation of mitochondrial cristae, as well as impaired mitochondrial biogenesis [45,46,47]. The inhibition of mitochondrial complexes by STZ is also widely discussed [48,49]. These data corroborate the decrease in TOMM20 immunofluorescence levels observed in the present study, which likely reflects a reduction in mitochondrial mass under the influence of STZ. On the other hand, in AD, defective mitochondria are known to accumulate in neurons in some cases, thereby increasing TOMM20 staining. This is attributed to impaired mitophagy in AD [50] and points to differences from the STZ model. Mitochondrial supply is fundamental for the functioning of the highly energy-dependent hippocampal synaptic connections, and the detected mitochondrial impairments are consistent with the observed decrease in synaptophysin staining.

Another possible cause of cognitive impairment in the STZ-induced AD model may be the increased sensitivity of developing hippocampal neurons to STZ, demonstrated both in vitro and in vivo [51,52], which is also consistent with our data. Interestingly, reduced neurogenesis, but not cell proliferation, is a delayed effect (3 months post-administration) of STZ and, according to Sun et al. [42], occurs predominantly in the septal (anterior) hippocampus. These findings align with our hypothesis that vimentin-expressing glia detected in the subgranular zone may originate from neuronal precursors and reflect a shift in their differentiation toward gliogenesis. The latter, in turn, could result from STZ-induced metabolic suppression and the greater resilience of astroglia to glucose metabolism disturbances.

STZ-induced reactive astrogliosis, demonstrated in our study by GFAP and vimentin staining, has also been reported in other STZ AD models [33] and correlates with significant increases in GFAP-positive glia and IBA1-microglia in human CA1-CA3 hippocampal fields in AD [53]. Astroglial activation and dysfunction may impair synaptic transmission modulation, induce excitotoxicity, increase ROS production, and reduce clearance of amyloid-β and tau protein aggregates typical for AD [53].

STZ administration appears to induce the formation of a rich spectrum of reactive astrocyte phenotypes in response to neuronal injury. Reactive astrogliosis is defined by GFAP upregulation, remodeling of astrocyte processes, and the retraction of astrocytic endfeet. It is also accompanied by altered AQP4 levels and its redistribution across the astrocytic membrane [54]. Depending on the reactive phenotype of the astrocytes, their response may involve either an increase or a decrease in AQP4 expression [55,56]. Reported direct STZ toxicity to astrocytes and endothelial cells [57] may suppress AQP4 expression and cause astroglial death, while also triggering proliferation and gliovascular remodeling.

In our study, we recorded a decrease in overall AQP4 immunofluorescence in the CA3 field of the hippocampus alongside an increase in GFAP staining intensity, and observed reactive astrocytes exhibiting AQP4 redistribution. These findings could be explained by both structural alterations in the neuropil—such as the reduction and thickening of astrocytic peripheral processes and by changes in the AQP4 regulation, which warrants further investigation.

Literature data regarding changes in AQP4 levels and distribution in the STZ model remain scarce, a limitation that was also emphasized in a recent review [58]. Certain inconsistencies exist within the current literature. For instance, a combined increase in AQP4 and GFAP levels was reported in STZ-treated rats 4 weeks post-administration [59]. In another experiment, STZ-treated mice exhibited an increase in GFAP levels, whereas AQP4 staining intensity remained unchanged, although the authors identified AQP4 redistribution [60]. Other studies indicate a local increase in AQP4 levels within glial cells surrounding tau and β-amyloid deposits in genetic models [61]. These variations likely reflect different phases of the reactive astrocyte response or the heterogeneity of their reactivity profiles shaped by distinct experimental conditions.

Our data demonstrate substantial remodeling of gliovascular interactions in the hippocampus following STZ administration and complement existing evidence that aquaporin expression levels and localization on astrocytic endfeet are altered during neuroinflammation in neurodegenerative disease models [42]. Postmortem human studies show that AD and aging are associated with disrupted AQP4 localization, while preserved perivascular AQP4 distribution correlates with the absence of cognitive impairment in senescent individuals [62,63]. However, it remains unclear whether amyloid-β drives AQP4 mislocalization or whether inflammatory processes and astrocytic water channel dysfunction promote amyloid-β accumulation in AD [64].

In agreement with an earlier report [65], the present study demonstrates that ventricular enlargement is among the consequences of STZ administration. This may occur not only as a secondary result of the atrophy of brain structures but also due to impaired dynamics and exchange of cerebrospinal and interstitial fluids. The latter is linked, in part, to the maintenance of water homeostasis by astrocytes, and these alterations are associated with the impaired clearance of pathological proteins. In patients with AD, reactive gliosis of the ventricular ependymal layer, ventriculomegaly, and periventricular edema are observed [66,67,68], which aligns with our findings.

Overall, considering the complex STZ-induced alterations and existing literature, we conclude that intracerebroventricular STZ administration represents an effective model of sporadic AD, recapitulating progressive morphological and behavioral changes.

### 4.2. Semaglutide Partially Attenuates Behavioral and Morphological Changes in an AD Model

Our findings demonstrate reduced tau protein phosphorylation and a trend toward less hippocampal neuron damage in the STZ model following semaglutide administration. These results align with the study by Wang et al. on the 3xTg genetic AD model [69]. Semaglutide increased the expression levels of sirtuin-1 (associated with insulin sensitivity regulation and mitochondrial biogenesis) and the glucose transporter GLUT4 in the hippocampus, accompanied by improved cognitive performance and reduced senile plaque burden [70]. These findings are consistent with an earlier study demonstrating semaglutide neuroprotective effects on SH-SY5Y neuroblastoma cells treated by amyloid-β peptide 25–35 [70]. However, conflicting data exist: Germano et al. [71] reported that semaglutide did not reduce thioflavin-stained amyloid plaques in 5xFAD and male APP/PS1 AD model mice, though it showed effects in females of the latter strain. The same study found no significant effects of semaglutide on microglial or astroglial activation. Conversely, another APP/PS1 mouse study revealed cognitive (but not motor) improvements interpreted as semaglutide anxiolytic effects [72].

In the NOR task, STZ-treated rats receiving semaglutide showed improved cognitive function compared to STZ-only controls. The STZ + semaglutide group demonstrated enhanced novel object discrimination, suggesting a potential mitigation of recognition and short-term memory deficits. This aligns with previous APP/PS1 mouse research, where GLP1R-agonist liraglutide increased novel object interaction time [44]. It is possible that the observed improvement in behavioral performance reflects an early restoration of synaptic function and neuroplasticity, which may precede detectable morphological changes at the structural level.

Numerous data support the role of the hippocampus in the NOR test, including its dorsal part [73,74]. However, these cited works emphasize the presence of mixed results. Such variability is observed across both lesion and inactivation studies of hippocampal circuits. Consequently, a direct causal link between CA3 area damage and decreased NOR performance in STZ-treated rats is difficult to assert, considering the widespread lesions caused by STZ.

At the same time, the role of GLP1R signaling in the recovery of cognitive functions is emphasized by data showing that receptor deficiency reduces synaptic plasticity and cognitive performance [75]. An age-dependent decline in GLP1 levels has been identified in humans, most pronounced in the neocortex and hippocampus [17], suggesting an association between reduced GLP1 levels and cognitive impairments and neurodegenerative diseases.

The effect of semaglutide on microglial activation demonstrated in this study complements previous findings on the conversion of its pro-inflammatory phenotype (M1) to a reparative phenotype (M2) observed in transgenic mice with the 3xTg AD model following semaglutide administration [69]. These results are further corroborated by studies in other models: reserpine-induced fibromyalgia [76] and middle cerebral artery (MCA) occlusion [77], where semaglutide reduced microglial activation and attenuated astrocytic expression of complement component C3, a marker of pro-inflammatory activation. Although no reduction in GFAP expression was observed in the STZ-induced AD model in this study, the restoration of AQP4 localization with semaglutide suggests diminished reactive astrogliosis. This observation aligns with the similar effect of the GLP1R agonist liraglutide in a genetic AD model (APP NL-G-F) and is supported by in vitro studies showing liraglutide modulation of AQP4 phosphorylation, which governs its localization to astrocytic endfeet [78]. Furthermore, GLP-1 agonists have been shown to reduce CSF secretion [79], which aligns with our results and may act as a neuroprotective factor. Semaglutide is likely to modulate the functioning of the glymphatic system, thereby improving cerebrospinal fluid exchange, which is consistent with the observed reduction in ventriculomegaly in the STZ animal model.

Given the role of glial cells in synaptic maintenance and clearance of pathological protein aggregates, our findings suggest that semaglutide could exert ameliorative effects that are largely associated with the suppression of glial proinflammatory reactions. Our findings of enhanced preservation of the synaptic marker in the hippocampus of semaglutide-treated animals with STZ-induced injury are consistent with recent reports demonstrating the drug’s ability to reduce microglial activation and synaptic phagocytosis in the hypothalamus of high-fat diet-fed rodents [80]. Notably, the proteolytic GLP1 fragment (9–36)—lacking insulinotropic activity—enhanced dendritic spine maturation in the hippocampus of a Down syndrome model (Ts65Dn) [81], suggesting insulin-independent GLP1-mediated effects on synaptogenesis.

Collectively, our data suggest that the semaglutide effects identified at the morphological level may be associated with the attenuation of behavioral changes in an intracerebroventricular STZ sporadic AD model.

## 5. Conclusions

A single course of semaglutide administration at the onset of neurodegeneration did not completely prevent the neurodegenerative process, although it primarily at a trend level attenuated STZ-induced alterations, including the reduction in hippocampal neuron count and mitochondrial staining in the CA3 field. Semaglutide showed no effect on STZ-impaired neurogenesis.

At the same time, semaglutide exerted certain beneficial prolonged effects on STZ-induced hippocampal injury—it mitigated the decrease in synaptophysin staining in the hippocampus and phospho-tau accumulation, attenuated microglial activation, reduced the number of vimentin-positive reactive astrocytes, and appeared to modulate AQP4 levels and distribution, as well as ventricular enlargement.

## Data Availability

The dataset is available on request from the authors.

## Abbreviations

GLP1	Glucagon-like peptide 1
GLP1R	Glucagon-like peptide 1 receptor
AD	Alzheimer disease
STZ	Streptozotocin
